# RETRACTION: The Effect of Incisional Negative Pressure Wound Therapy on the Improvement of Postoperative Cosmetic Suture Wounds and Scar Hyperplasia

**DOI:** 10.1111/iwj.70303

**Published:** 2025-03-06

## Abstract

**RETRACTION:** LiuY. , XuM. , WangZ. , ZhuX. , and XuJ. , “The Effect of Incisional Negative Pressure Wound Therapy on the Improvement of Postoperative Cosmetic Suture Wounds and Scar Hyperplasia,” International Wound Journal 20, no. 8 (2024): 3081–3087, 10.1111/iwj.14183.PMC1050224237114415

The above article, published online on 28 April 2023, in Wiley Online Library (http://onlinelibrary.wiley.com/), has been retracted by agreement between the journal Editor in Chief, Professor Keith Harding; and John Wiley & Sons Ltd. Following an investigation by the publisher, all parties have concluded that this article was accepted solely on the basis of a compromised peer review process. The editors have therefore decided to retract the article. The authors did not respond to our notice regarding the retraction.

**RETRACTION:** Y. Liu , M. Xu , Z. Wang , X. Zhu , and J. Xu , “The Effect of Incisional Negative Pressure Wound Therapy on the Improvement of Postoperative Cosmetic Suture Wounds and Scar Hyperplasia,” International Wound Journal 20, no. 8 (2024): 3081–3087, 10.1111/iwj.14183.PMC1050224237114415

The above article, published online on 28 April 2023, in Wiley Online Library (http://onlinelibrary.wiley.com/), has been retracted by agreement between the journal Editor in Chief, Professor Keith Harding; and John Wiley & Sons Ltd. Following an investigation by the publisher, all parties have concluded that this article was accepted solely on the basis of a compromised peer review process. The editors have therefore decided to retract the article. The authors did not respond to our notice regarding the retraction.

